# The Complete Loss of p53 Expression Uniquely Predicts Worse Prognosis in Colorectal Cancer

**DOI:** 10.3390/ijms23063252

**Published:** 2022-03-17

**Authors:** Kazuhiro Nagao, Akira Koshino, Akane Sugimura-Nagata, Aya Nagano, Masayuki Komura, Akane Ueki, Masahide Ebi, Naotaka Ogasawara, Toyonori Tsuzuki, Kenji Kasai, Satoru Takahashi, Kunio Kasugai, Shingo Inaguma

**Affiliations:** 1 Division of Gastroenterology, Department of Internal Medicine, Aichi Medical University School of Medicine, Nagakute 480-1195, Japan; nagao.kazuhiro.621@mail.aichi-med-u.ac.jp (K.N.); koshino.akira.255@mail.aichi-med-u.ac.jp (A.K.); nagata.akane.240@mail.aichi-med-u.ac.jp (A.S.-N.); ebi.masahide.814@mail.aichi-med-u.ac.jp (M.E.); ogasawara.naotaka.667@mail.aichi-med-u.ac.jp (N.O.); kasugai.kunio.527@mail.aichi-med-u.ac.jp (K.K.); 2 Department of Experimental Pathology and Tumor Biology, Nagoya City University Graduate School of Medical Sciences, Nagoya 467-8601, Japan; aya.ngn@med.nagoya-cu.ac.jp (A.N.); komura@med.nagoya-cu.ac.jp (M.K.); c211705@ed.nagoya-cu.ac.jp (A.U.); sattak@med.nagoya-cu.ac.jp (S.T.); 3 Department of Surgical Pathology, Aichi Medical University School of Medicine, Nagakute 480-1195, Japan; tsuzuki@aichi-med-u.ac.jp; 4 Department of Pathology, Aichi Medical University School of Medicine, Nagakute 480-1195, Japan; kkasai@aichi-med-u.ac.jp; 5 Department of Pathology, Nagoya City University East Medical Center, Nagoya 464-8547, Japan

**Keywords:** colorectal cancer (CRC), immunohistochemistry, p53, CDX2, ALCAM, stem-like immunophenotype

## Abstract

p53 immunohistochemistry is considered an accurate surrogate marker reflecting the underlying *TP53* mutation status and has utility in tumor diagnostics. In the present study, 269 primary CRCs were immunohistochemically evaluated for p53 expression to assess its utility in diagnostic pathology and prognostication. p53 expression was wild-type in 59 cases (23%), overexpressed in 143 cases (55%), completely lost in 50 cases (19%), and cytoplasmic in 10 cases (4%). p53 immunoreactivity was associated with tumor size (*p* = 0.0056), mucus production (*p* = 0.0015), and mismatch repair (MMR) system status (*p* < 0.0001). Furthermore, among CRCs with wild-type p53 expression, a significantly higher number of cases had decreased CDX2 than those with p53 overexpression (*p* = 0.012) or complete p53 loss (*p* = 0.043). In contrast, among CRCs with p53 overexpression, there were significantly fewer ALCAM-positive cases than p53 wild-type cases (*p* = 0.0045). However, no significant association was detected between p53 immunoreactivity and the “stem-like” immunophenotype defined by CDX2 downregulation and ALCAM-positivity. Multivariate Cox hazards regression analysis identified tubular-forming histology (hazard ratio [HR] = 0.17, *p* < 0.0001), younger age (HR = 0.52, *p* = 0.021), and female sex (HR = 0.55, *p* = 0.046) as potential favorable factors. The analysis also revealed complete p53 loss (HR = 2.16, *p* = 0.0087), incomplete resection (HR = 2.65, *p* = 0.0068), and peritoneal metastasis (HR = 5.32, *p* < 0.0001) as potential independent risk factors for patients with CRC. The sub-cohort survival analyses classified according to chemotherapy after surgery revealed that CRC patients with wild-type p53 expression tended to have better survival than those with overexpression or complete loss after chemotherapy. Thus, immunohistochemistry for p53 could be used for the prognostication and chemotherapy target selection of patients with CRC.

## 1. Introduction

Multiple biomarkers have been identified to assist in disease diagnosis and predict treatment efficacy and patient outcomes for cancers such as colorectal cancer (CRC) [1]. Recently, our group established a tissue-microarray-based cohort to explore prognostic markers, and successfully identified several prognostic markers predicting both favorable and unfavorable clinical outcomes in CRC patients [2,3,4,5,6].

The tumor suppressor p53 was first identified in 1979 as an oncoprotein [7,8,9]. Numerous studies have since been performed to clarify the function of p53. The essential functions and characteristics of p53 are considered to be as follows: (1) p53 is a transcription factor that activates its specific target genes [10]; (2) p53 exerts tumor-suppressive effects through cell cycle arrest, apoptosis, DNA repair, and anti-angiogenesis [11]; (3) suppressed p53 levels have an important role in the maintenance of stem cell and cancer stem cell phenotypes [12,13]; and (4) p53 is one of the most mutated genes in a broad range of human cancers [14]. Most *TP53* mutations are single missense mutations within its DNA-binding domain which lead to a large spectrum of functional effects. Mutations in *TP53* result in different isoforms with variable transcriptional activity that result in different cancer phenotypes [15].

p53 immunohistochemistry is considered to be an accurate surrogate marker that reflects the underlying mutational status of *TP53* and has utility in the diagnostics of tumors [16]. It has long been recognized that nonsynonymous *TP53* missense mutations result in the nuclear accumulation of p53 protein that can be detected as overexpression in the form of diffuse strong nuclear positivity involving at least 80% of the tumor cells, but usually almost 100% [16]. Furthermore, other abnormal p53 expression patterns, cytoplasmic expression [17] and complete loss [18], have been recognized to correlate with the presence of a *TP53* mutation.

Stem cell markers such as ALCAM (activated leukocyte cell adhesion molecule, CD166), ALDH1A1 (aldehyde dehydrogenase 1 family member A1, ALDH1), and SALL4 (spalt-like transcription factor 4) have been reported to be variably expressed in CRCs and linked to unfavorable clinical outcomes [19,20,21,22]. More recently, expression of CDX2 was found to be inversely correlated with ALCAM expression. Furthermore, the loss of CDX2 expression has been shown to be a poor prognostic indicator in stage II and stage III colon carcinoma [23]. Through immunohistochemical analyses, our group identified a significant association between CD274 (PD-L1) expression and a “stem-like” immunophenotype defined by the loss or weak expression of CDX2 and ALCAM positivity in American and European populations. However, the prognostic significance of this “stem-like” immunophenotype was not evaluated because of the lack of the survival information in that cohort [24].

The present study immunohistochemically examined the expression status of p53 in CRCs. The associations of p53 immunoreactivity with clinicopathological features and clinical outcomes were analyzed to assess their potential for clinical use. In addition, the associations between p53 and cellular proliferation markers or the “stem-like” immunophenotype were analyzed to characterize CRCs with different p53 immunoreactivity patterns.

## 2. Results

### 2.1. Expression Status of p53 in CRCs

**Figure 1:**
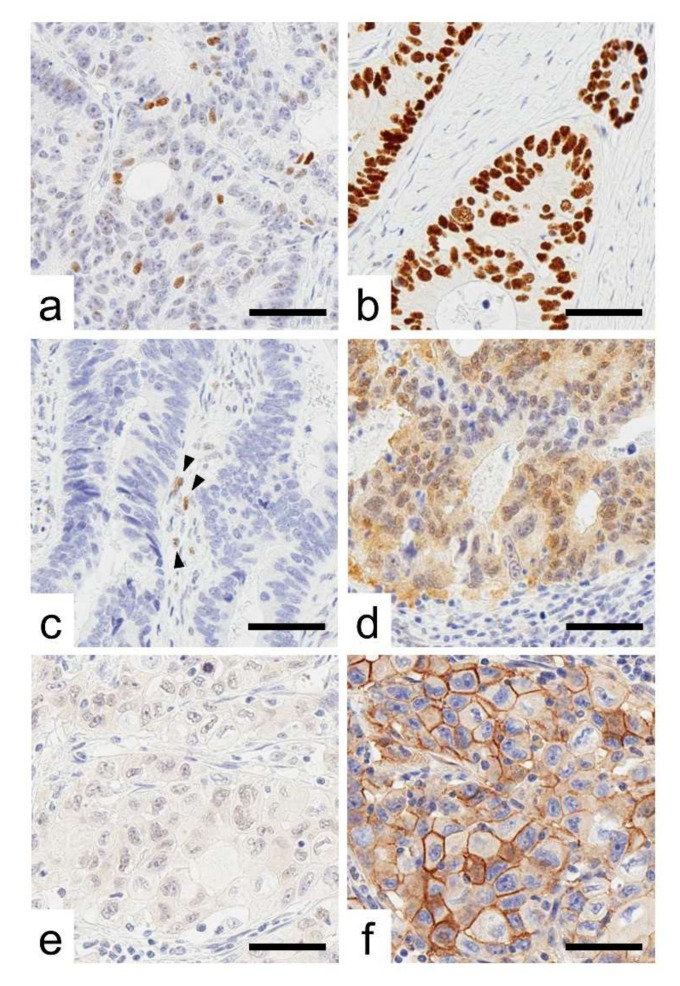
Representative images of p53 immunoreactivity. (**a**–**d**), Representative images of p53 immunoreactivity. (**a**) Wild-type pattern, (**b**) overexpression, (**c**) cytoplasmic expression, and (**d**) complete loss. Arrow heads indicate internal control cells with weak p53 nuclear expression. (**e**,**f**), CRC cases with the “stem-like” immunophenotype showing CDX2-negative (**e**) and ALCAM-positive expression (**f**). Bar, 50 µm.

Representative images for CRC cases with immunoreactivity for p53, lost CDX2, and ALCAM are presented in Figure 1. p53 immunoreactivity was observed as follows: wild-type pattern (59 cases, 23%), overexpression (143 cases, 55%), complete loss (50 cases, 19%), and cytoplasmic expression (10 cases, 4%). The clinical, pathological, and immunohistochemical features of the analyzed tumors are summarized in Table 1 according to p53 immunoreactivity. p53 immunoreactivity was associated with tumor size.

**Figure 2:**
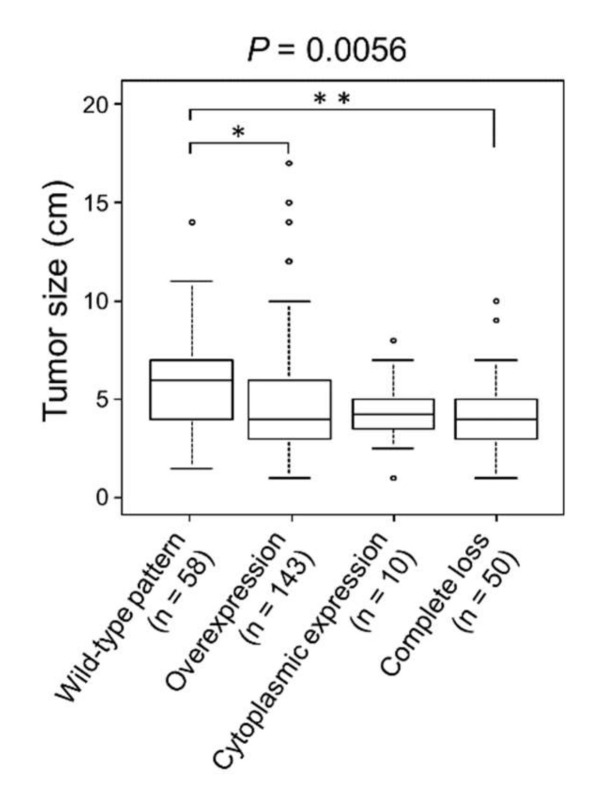
Association of p53 immunoreactivity and tumor size. CRCs with wild-type p53 expression showed significantly larger tumors than CRCs with p53 overexpression or complete loss. * *p* < 0.05; ** *p* < 0.01.

CRCs with wild-type pattern p53 expression showed significantly larger size than overexpression (*p* = 0.030) or completely lost type (*p* = 0.0037) of CRCs (Table 1 and Figure 2).

Cases with wild-type p53 immunoreactivity contained a significantly higher number of mucus-producing (*p* = 0.0015) and mismatch repair (MMR) system-deficient CRCs (*p* < 0.0001).

Table 2 presents the results of CDX2 and ALCAM expressions and “Stem-like” immunophenotype according to p53 immunoreactivity. Weak or negative expression of CDX2 was seen in 11% (29/233 cases) of CRCs. CRCs with p53 wild-type expression contained significantly higher numbers of CDX2 decreased cases than p53 overexpression (*p* = 0.012) or complete loss cases (*p* = 0.043). In contrast, ALCAM was expressed in 29% (76/262 cases). CRCs with p53 overexpression contained significantly lower numbers of ALCAM-positive cases than p53 wild-type (*p* = 0.0045). No significant association was detected between “stem-like” immunophenotype and p53 immunoreactivity.

### 2.2. p53 Immunoreactivity Correlated with PHH3 Expression

**Figure 3:**
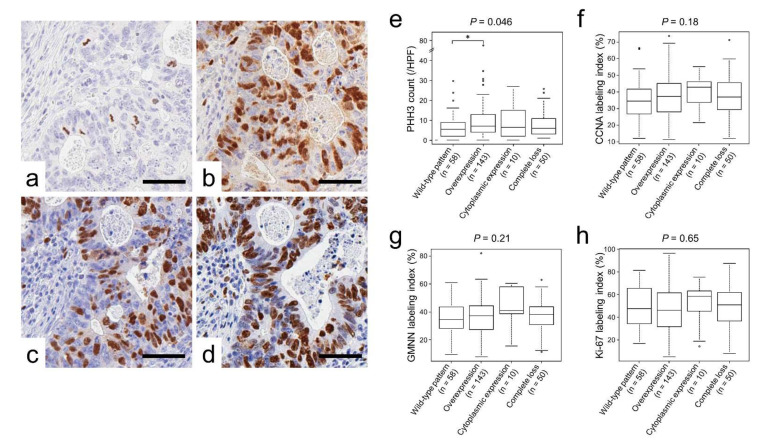
Cellular proliferation marker expression classified according to p53 expression. (**a**–**d**), representative images of immunohistochemistry for cellular proliferation markers. (**a**) PHH3, (**b**) CCNA, (**c**) GMNN, and (**d**) Ki-67. (**e**–**h**) Cellular proliferation marker expression classified by p53 immunoreactivity. (**e**) PHH3, (**f**) CCNA, (**g**) GMNN, and (**h**) Ki-67. Note that p53-overexpressing tumors contained a significantly higher number of PHH3-positive cells than wild-type tumors. * *p* < 0.05.

Table 3 and Figure 3e–h to h show the associations between p53 immunoreactivity and cellular proliferation marker expression. Immunohistochemical staining analyses revealed a unique association between p53 immunoreactivity and phospho-histone H3 (PHH3)-positive cell counts (*p* = 0.046; Table 3 and Figure 3e). In contrast, no significant difference was detected between p53 expression and cyclin A (CCNA), geminin (GMNN), or Ki-67 labeling indices.

### 2.3. Survival Analyses of Patients with CRC According to p53 Immunoreactivity

**Figure 4:**
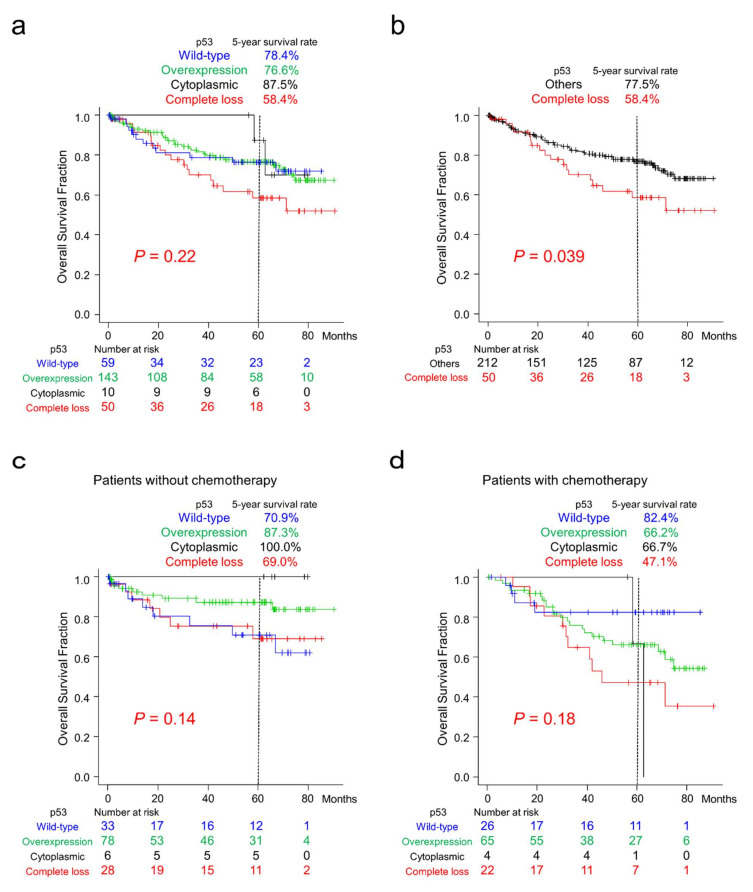
Overall survival of patients with CRC classified according to p53 immunoreactivity. (**a**) Kaplan–Meier curves for patients classified according to the four p53 expression patterns. CRC patients with complete loss of p53 tended to show worse survival. (**b**) Kaplan–Meier curves for patients classified into two groups according to p53 immunoreactivity. Note that CRC patients with complete loss of p53 showed significantly worse survival than the group with the other three expression patterns. (**c**) Kaplan–Meier curves for patients without chemotherapy classified according to four different p53 expression patterns. CRC patients with wild-type p53 expression tended to show worse survival than the others. (**d**) Kaplan–Meier curves for patients with post-surgery chemotherapy classified according to p53 immunoreactivity. CRC patients with wild-type p53 expression tended to show more favorable survival than the group with p53 overexpression or complete loss.

Among the four patterns of p53 immunoreactivity, CRC patients with complete loss of p53 tended to show worse clinical outcomes (Figure 4a). After reclassification according to p53 immunoreactivity, patients with complete loss of p53 CRC showed significantly worse clinical outcomes than those with the other three types of immunoreactivity (Figure 4b). p53-overexpressing cases tended to show better survival than the other groups after reclassification (Supplementary Figure S1).

The results of sub-cohort survival analyses classified according to chemotherapy after surgery are shown in Figure 4c,d. In patients without chemotherapy after resection, patients with CRC exhibiting wild-type p53 immunoreactivity tended to show worse survival than the other groups (Figure 4c). In contrast, these patients tended to show better survival than patients with p53 overexpression or complete loss in the sub-cohort with chemotherapy (Figure 4d).

Multivariate Cox hazards regression analysis identified tubular-forming histology (hazard ratio [HR] = 0.17, 95% confidence interval [CI] = 0.09–0.33, *p* < 0.0001), younger age (<70 years old, HR = 0.52, 95% CI = 0.30–0.91, *p* = 0.021), and sex (female, HR = 0.55, 95% CI = 0.31–0.99, *p* = 0.046) as potential favorable factors. The analysis also revealed complete loss of p53 expression (HR = 2.16, 95% CI = 1.21–3.86, *p* = 0.0087), incomplete resection (HR = 2.65, 95% CI = 1.31–5.35, *p* = 0.0068), and peritoneal metastasis (HR = 5.32; 95% CI = 2.97–9.54, *p* < 0.0001) as potential independent risk factors for patients with CRC (Table 4).

### 2.4. Survival Analyses of Patients with CRC According to CDX2 and ALCAM Expression

CRC cases with decreased CDX2 expression (*p* < 0.0001) and cases with the “stem-like” immunophenotype (*p* = 0.0027) showed significantly worse clinical outcomes (Supplementary Figure S2a,c). ALCAM expression had no correlation with the survival of CRC patients (*p* = 0.44; Supplementary Figure S2b).

## 3. Discussion

Prognostication using p53 immunohistochemistry has been undertaken in many cancer types, including CRC [25,26,27,28]. Most of the initial studies focused on nuclear p53 overexpression and divided the cohorts into two groups, overexpression and lower expressors, and most found unfavorable clinical outcomes in the former [26]. In our cohort, p53-overexpressing cases tended to show better clinical outcomes than the other groups, but no significant difference was detected (*p* = 0.29; Supplementary Figure S1). Based on the improved sensitivity and specificity of immunohistochemistry by the development of automated immunostainer and high-quality antibodies, it now allows us to distinguish faint p53 expression patterns of wild-type, complete loss, and some cases of cytoplasmic expression patterns. Although using a four-group analysis also failed to detect significant differences (Figure 4a), reclassification successfully identified significantly worse clinical outcomes in cases with complete loss (*p* = 0.039; Figure 4b). Analysis of *TP53* expression data from The Cancer Genome Atlas (TCGA) also revealed that patients with lower *TP53*-expressing CRC tended to show unfavorable survival (*p* = 0.17; Supplementary Figure S3a); however, it was considered that *TP53* expression levels and p53 immunoreactivity would not be parallel. Patients with *TP53*-mutant CRCs also tended to show unfavorable clinical outcomes (*p* = 0.064; Supplementary Figure S3b). On the basis of the large spectrum of cancer phenotypes resulting from the many mutations in *TP53* [15], gene-mutation-specific classification may identify a better prognostication system.

CRCs are classified into four consensus molecular subtypes (CMSs) with distinguishing features: CMS1 (microsatellite instability immune type), hypermutated, microsatellite unstable, and strong immune activation; CMS2 (canonical type), epithelial, and marked WNT and MYC signaling activation; CMS3 (metabolic type), epithelial and evident metabolic dysregulation; and CMS4 (mesenchymal type), prominent transforming growth factor activation, stromal invasion, and angiogenesis [29]. In the present study, among CRCs with wild-type p53 expression, there was a significantly higher number of MMR-deficient cases, at 32% (19/59 cases). Conversely, 63% (19/30 cases) of MMR-deficient cases showed the wild-type p53 expression pattern. These observations were consistent with a previous report that CMS1-positive CRCs harbor the rarest *TP53* mutations among the four groups [29].

Suppressed p53 levels play an important role in stem cell maintenance, cancer stem cell phenotypes, induced pluripotent stem cells, and other stem cell roles and behaviors [12,13]. CRC stem cells characterized by the expression of markers such as ALCAM have been reported to harbor tumor-initiating and highly proliferative potential in tumor xenograft models [20,30]. Furthermore, high stem cell marker expression in the CRC cells of surgically resected specimens have been reported to show worse clinical outcomes [19,20,21,22]. To the best of our knowledge, the associations of p53 and stemness-related gene expressions, such as CDX2 and ALCAM, have never been immunohistochemically evaluated in CRCs. In the present study, the association of p53 immunoreactivity and the stem-like immunophenotype, defined by decreased CDX2 and positive expression of ALCAM [24], was analyzed. Intriguingly, among wild-type p53-expressing CRCs, there was a significantly higher number of cases with downregulated CDX2 (*p* = 0.0031). Furthermore, p53-overexpressing CRCs included a significantly lower number of ALCAM-positive tumors (*p* = 0.0016). However, no correlation between p53 immunoreactivity and the “stem-like” immunophenotype was detected (*p* = 0.19). This observation might be because of the very low number of cases with the “stem-like” immunophenotype (4%, 10/262 cases). In our previous study, the prognostic significance of CDX2, ALCAM, or the “stem-like” immunophenotype was not analyzed because of the lack of prognostic data [24]. In the present study, prognostic analyses identified significantly worse clinical outcomes in CDX2-downregulated or “stem-like” immunophenotype-positive CRCs. Our observations, in part, are in line with previous reports demonstrating the poor prognosis of CRC cases with loss of CDX2 [19,23].

PHH3, which is expressed during late G2 and M, is a specific immunohistochemical indicator of proliferating cells in FFPE sections. Furthermore, in diagnostic pathology, the immunohistochemical detection of PHH3 is useful for grading, prognostication, and assessment of recurrence risk, and as an indicator of response to treatment [31,32,33,34,35]. In the present study, we were intrigued to identify unique and significant differences in PHH3 expression between CRCs with wild-type p53 expression and p53 overexpression (Figure 3e and Table 3). To the best of our knowledge, the association of PHH3 and p53 expressions in carcinomas including CRC has never been reported. Cell cycle arrest and apoptosis are the most notable biological outcomes of p53 activation. This observation may indicate the preserved cell cycle arrest function of p53 in tumors with wild-type p53 expression. However, it is unclear why wild-type p53 expression resulted in the largest tumors (Table 1 and Figure 2).

Chemotherapy-activated p53 can induce apoptotic cell death in cancer cells [26]. Therefore, we hypothesized that CRC patients classified according to p53 immunoreactivity might show differential reactions to chemotherapy administered after resection. In our sub-cohort analysis of patients who did not undergo chemotherapy after surgery (*n* = 145), those with wild-type p53 expression tended to show worse survival (Figure 4c). In contrast, in the sub-cohort of patients treated with chemotherapy after surgery (*n* = 117), as noted previously [26], CRC patients with wild-type p53 expression tended to show better survival than those with p53 overexpression or complete loss (Figure 4d). These results suggest that CRC patients with wild-type p53 expression might be good targets for systemic chemotherapy because of the preserved apoptotic inducibility of p53. Recently, ribosomal proteins have been shown to partly regulate chemotherapy-induced cellular responses either in a p53-dependent or p53-independent manner [36]. Further expression analyses of ribosomal proteins may improve our understanding of chemosensitivity in CRCs.

The limitations of this study are the number of patients and the duration of follow-up. As shown in Figure 4c,d, log-rank tests analyzing sub-cohorts classified according to chemotherapy after surgery failed to identify a significant difference between groups classified by p53 immunoreactivity. Furthermore, no correlation between p53 immunoreactivity and the “stem-like” immunophenotype was detected (*p* = 0.19). A larger cohort with a longer follow-up duration may optimize prognostication models and identify important characteristics of CRCs.

The present study immunohistochemically evaluated the expression of p53 in CRCs. CRC patients with complete loss of p53 showed significantly worse clinical outcomes. Furthermore, CRC patients with wild-type p53 expression might be good targets for systemic chemotherapy. Immunohistochemistry for p53 could be used for the prognostication and chemotherapy target selection of patients with CRC even in those hospitals that do not have gene sequencing facilities.

## 4. Materials and Methods

### 4.1. Tissue Samples

The Institutional Ethical Review Board of Aichi Medical University Hospital permitted this project to be performed without patient consent by providing the opportunity for opt-out. A total of 269 formalin-fixed, paraffin-embedded (FFPE) samples of primary colorectal tumors resected at Aichi Medical University Hospital from 2009 to 2012 were collected depending on the availability of tissue samples and clinical information. After surgery, patients were followed up for up to 90 months. All tumors were diagnosed as invasive and naïve to chemotherapy or radiotherapy. Staging of tumors was performed according to the TNM classification of malignant tumors, eighth edition [37]. Tumors with glandular formation (>50%) or mucus production (>50% of the area) were defined as having differentiated or mucus-producing histology. A single 4.5 mm core tumor tissue sample derived from an FFPE specimen was assembled into multitumor blocks containing up to 30 samples. All cores were obtained from invasive areas, and approximately 20% of cores contained an invasive front. Non-neoplastic colonic mucosa adjacent to the tumor was also immunohistochemically analyzed.

### 4.2. Immunohistochemistry

The antibodies used in the present study are summarized in Supplementary Table S1. Immunohistochemistry was performed using a Leica Bond-Max (Leica Biosystems, Wetzlar, Germany) or Ventana BenchMark XT automated immunostainer (Roche Diagnostics, Basel, Switzerland). Signals were visualized using 3,3′-diaminobenzidine.

p53 immunoreactivity was classified as follows: wild-type, overexpression, complete loss, and cytoplasmic expression [16]. In the evaluation of complete loss of p53 expression, cases without internal controls such as fibroblasts and lymphoid cells were eliminated from the study. CDX2 and ALCAM immunoreactivity was classified according to our previous study: CDX2, positive vs. decreased or lost; and ALCAM, >5% on the cytomembrane of tumor cells [24]. The “stem-like” immunophenotype was defined by the loss or decreased expression of CDX2 as well as ALCAM positivity [24].

The data for cellular proliferation markers were cited from our previous study [4]. In brief, Ki-67, CCNA, and GMNN labeling indices were determined by counting >500 tumor cells per case in a high-power field (×400). The number of PHH3-positive cells was counted using the same magnification.

### 4.3. Statistical Analyses

Statistical analyses were performed using EZR software version 1.41 [38]. Fisher’s exact test or Kruskal–Wallis test with post hoc analyses was performed to analyze the statistical correlations between categorical data. Simple Bonferroni correction for multiple hypothesis testing was applied for adjustment at a two-sided alpha level of 0.0042 (=0.05/12).

For survival analyses, Kaplan–Meier survival estimates were calculated with the log-rank test. Cox proportional hazards regression analysis was performed to analyze the associations of survival with other factors. The initial model comprised sex (male vs. female), age (<70 years old vs. ≥70 years old), tumor size (<5 cm vs. ≥5 cm), primary tumor location (right-sided colon vs. left-sided colon vs. rectum), pT stage (pT2 vs. pT3 vs. pT4), tumor histology (moderate to well-differentiated vs. poorly differentiated), mucus production (positive vs. negative), lymph node metastasis (positive vs. negative), peritoneal metastasis (positive vs. negative), distant organ metastasis (positive vs. negative), surgical status (complete vs. incomplete resection), mismatch repair system status (preserved vs. deficient), and immunohistochemical data (p53 complete loss vs. others). A backward elimination with a threshold of *p* < 0.05 was used to select the variables in the final model.

## Figures and Tables

**Table 1:** Association between p53 immunoreactivity and clinicopathological features.

	Total No.		p53 Immunoreactivity					
	262	(100%)	Wild-Type 59 (23%)	Overexpression 143 (55%)	Cytoplasmic Expression 10 (4%)	Complete Loss 50 (19%)	*p*-Value	
Sex							0.49	a
Male	139	[53%]	36 [61%]	73 [51%]	4 [40%]	26 [52%]		
Female	123	[47%]	23 [39%]	70 [49%]	6 [60%]	24 [48%]		
Age, years (Mean ± S.D.)	68.6 ± 12.6		68.5 ± 14.1	68.5 ± 12.0	70.8 ± 7.7	69.0 ± 12.9	0.79	b
Size, cm (Mean ± S.D.)	5.0 ± 2.6		5.9 ± 2.6 †	4.9 ± 2.7	4.5 ± 1.9	4.3 ± 1.8	0.0056	b
Tumor location							0.10	c
Right-sided colon	120	[46%]	35 [59%]	58 [40%]	2 [20%]	25 [50%]		
Left-sided colon	84	[32%]	16 [27%]	51 [36%]	5 [50%]	12 [24%]		
Rectum	58	[22%]	8 [14%]	34 [24%]	3 [30%]	13 [26%]		
pT stage							0.31	a
pT2	36	[13%]	8 [14%]	18 [13%]	1 [10%]	9 [18%]		
pT3	182	[70%]	42 [71%]	103 [72%]	9 [90%]	28 [56%]		
pT4	44	[16%]	9 [15%]	22 [15%]	0 [0%]	13 [26%]		
Histological differentiation							0.73	a
Well to moderately	235	[90%]	51 [86%]	129 [90%]	10 [100%]	45 [90%]		
Poorly	27	[10%]	8 [14%]	14 [10%]	0 [0%]	5 [10%]		
Mucus production							0.0015	a
Positive	14	[5%]	9 [15%] ††	2 [1%]	0 [0%]	3 [6%]		
Negative	248	[95%]	50 [85%]	141 [99%]	10 [100%]	47 [94%]		
Lymph node metastasis							0.19	a
Positive	122	[49%]	21 [38%]	67 [51%]	5 [50%]	29 [59%]		
Negative	124	[51%]	34 [62%]	65 [49%]	5 [50%]	20 [41%]		
Peritoneal metastasis								a
Positive	49	[19%]	7 [12%]	32 [22%]	0 [0%]	10 [20%]	0.15	
Negative	213	[81%]	52 [88%]	111 [78%]	10 [100%]	40 [80%]		
Distant organ metastasis								a
Positive	43	[16%]	5 [8%]	28 [20%]	0 [0%]	10 [20%]	0.11	
Negative	219	[84%]	54 [92%]	115 [80%]	10 [100%]	40 [80%]		
Operation status							0.23	a
Complete resection	230	[88%]	56 [95%]	122 [85%]	9 [90%]	43 [86%]		
Incomplete resection	32	[12%]	3 [5%]	21 [15%]	1 [10%]	7 [14%]		
MMR system status								a
Deficient	30	[12%]	19 [32%] †††	8 [6%]	1 [10%]	2 [4%]	<0.0001	
Preserved	232	[88%]	40 [68%]	135 [94%]	9 [90%]	48 [96%]		

a, Fisher’s exact, b, Kruskal–Wallis or c, chi-squared test with post hoc test (Holm) was used to calculate *p*-values. †: *p* = 0.030 vs. overexpression, *p* = 0.0037 vs. complete loss. ††: *p* = 0.0019 vs. overexpression. †††: *p* < 0.0001 vs. overexpression or complete loss. Data are shown as the median (25th, 75th percentiles). The Bonferroni-corrected *p*-value for significance was *p* ≈ 0.0042 (0.05/12).

**Table 2:** Association between p53 immunoreactivity and stem-like features.

	Total No.		p53 Immunoreactivity				
	262	(100%)	Wild-Type 59 (23%)	Overexpression 143 (55%)	Cytoplasmic Expression 10 (4%)	Complete Loss 50 (19%)	*p*-Value
CDX2							0.0031
Positive	233	[89%]	44 [75%] †	132 [92%]	10 [100%]	47 [94%]	
Decreased or lost	29	[11%]	15 [25%]	11 [8%]	0 [0%]	3 [6%]	
ALCAM							0.0016
Positive	76	[29%]	26 [44%]	28 [21%] ††	3 [30%]	19 [38%]	
Negative	186	[71%]	33 [56%]	115 [79%]	7 [70%]	31 [62%]	
Stem-like immunophenotype							0.19
Positive	10	[4%]	5 [8%]	3 [2%]	0 [0%]	2 [4%]	
Negative	252	[96%]	54 [92%]	140 [98%]	10 [100%]	48 [96%]	

Fisher’s exact test with post hoc test (Holm) was used to calculate *p*-values. †: *p* = 0.043 vs. complete loss, *p* = 0.012 vs. overexpression. ††: *p* = 0.0045 vs. wild-type.

**Table 3:** Association between p53 immunoreactivity and cellular proliferation marker expressions.

	Total No.	p53 Immunoreactivity				
	262 (100%)	Wild-Type 59 (23%)	Overexpression 143 (55%)	Cytoplasmic Expression 10 (4%)	Complete Loss 50 (19%)	*p*-Value
PHH3 (/HPF)	7.0 (3, 12)	5.0 (2.0, 9.0) †	7.0 (4.0, 13.0)	6.5 (2.3, 14.0)	6.0 (3.3, 11.0)	0.046
CCNA (%)	36.4 (28.3, 44.7)	34.3 (26.8, 40.9)	37.3 (28.0, 45.2)	42.8 (35.7, 46.0)	37.0 (29.6, 45.4)	0.18
GMNN (%)	37.1 (28.4, 43.7)	34.4 (27.7, 43.4)	37.2 (27.3, 44.3)	40.9 (38.7, 54.9)	38.2 (30.0, 43.6)	0.21
Ki-67 (%)	49.2 (34.2, 62.0)	48.3 (34.9, 65.5)	46.0 (31.6, 61.5)	58.5 (47.6, 62.6)	50.9 (36.5, 61.7)	0.65

Kruskal–Wallis with post hoc test (Holm) was used to calculate *p*-values. †: *p* = 0.031 vs. overexpression. Data are shown as the median (25th, 75th percentiles).

**Table 4:** Multivariable Cox hazards analysis of colorectal cancer patients.

	Hazard	95% CI		
	Ratio	Min	Max	*p*-Value
Well to moderately differentiated histology	0.17	0.09	0.33	<0.0001
Age (<70)	0.52	0.30	0.91	0.021
Sex (female)	0.55	0.31	0.99	0.046
p53 complete loss	2.16	1.21	3.86	0.0087
Incomplete resection	2.65	1.31	5.35	0.0068
Peritoneal metastasis	5.32	2.97	9.54	<0.0001

The multivariable Cox hazards analysis model initially included sex, age, primary tumor location, tumor size, pT stage, surgical status, tumor histology, mucus production, lymph node metastasis, distant organ metastasis, peritoneal metastasis, mismatch repair system status, and immunoreactivity for p53 (Complete loss vs. others). A backward elimination with a threshold of *p* = 0.05 was used to select variables in the final model.

## Data Availability

The datasets used and/or analyzed during the present study are available from the corresponding author on reasonable request.

## Supplementary Materials

The following are available online at https://www.mdpi.com/article/10.3390/ijms23063252/s1.

## References

[1] Oh H.-H., Joo Y.-E. (2020). Novel biomarkers for the diagnosis and prognosis of colorectal cancer. Intest. Res..

[2] Inoue S., Ito H., Tsunoda T., Murakami H., Ebi M., Ogasawara N., Kasugai K., Kasai K., Ikeda H., Inaguma S. (2019). CD70 expression in tumor-associated fibroblasts predicts worse survival in colorectal cancer patients. Virchows Arch..

[3] Inoue S., Tsunoda T., Riku M., Ito H., Inoko A., Murakami H., Ebi M., Ogasawara N., Pastan I., Kasugai K. (2020). Diffuse mesothelin expression leads to worse prognosis through enhanced cellular proliferation in colorectal cancer. Oncol. Lett..

[4] Koshino A., Inoue S., Sugimura-Nagata A., Nishiyama T., Murakami H., Ito H., Riku M., Inoko A., Ebi M., Ogasawara N. (2021). High phospho-histone H3 expression uniquely predicts favorable survival among four markers of cellular proliferation in colorectal cancer. Pathol. Int..

[5] Nagano-Matsuo A., Inoue S., Koshino A., Ota A., Nakao K., Komura M., Kato H., Naiki-Ito A., Watanabe K., Nagayasu Y. (2021). PBK expression predicts favorable survival in colorectal cancer patients. Virchows Arch..

[6] Sugimura-Nagata A., Koshino A., Inoue S., Matsuo-Nagano A., Komura M., Riku M., Ito H., Inoko A., Murakami H., Ebi M. (2021). Expression and Prognostic Significance of CD47-SIRPA Macrophage Checkpoint Molecules in Colorectal Cancer. Int. J. Mol. Sci..

[7] Lane D., Crawford L.V. (1979). T antigen is bound to a host protein in SV40-transformed cells. Nature.

[8] Linzer D.I., Levine A.J. (1979). Characterization of a 54K dalton cellular SV40 tumor antigen present in SV40-transformed cells and uninfected embryonal carcinoma cells. Cell.

[9] Kress M., May E., Cassingena R., May P. (1979). Cassingena and May. Simian virus 40-transformed cells express new species of proteins precipitable by anti-simian virus 40 tumor serum. J. Virol..

[10] Menendez D., Inga A., Resnick M.A. (2009). The expanding universe of p53 targets. Nat. Rev. Cancer.

[11] Vogelstein B., Lane D., Levine A.J. (2000). Surfing the p53 network. Nature.

[12] Jain A.K., Allton K., Iacovino M., Mahen E., Milczarek R.J., Zwaka T.P., Kyba M., Barton M.C. (2012). p53 regulates cell cycle and microRNAs to promote differentiation of human embryonic stem cells. PLoS Biol..

[13] Marión R.M., Strati K., Li H., Murga M., Blanco R., Ortega S., Fernandez-Capetillo O., Serrano M., Blasco M.A. (2009). A p53-mediated DNA damage response limits reprogramming to ensure iPS cell genomic integrity. Nature.

[14] Hollstein M., Sidransky D., Vogelstein B., Harris C.C. (1991). p53 mutations in human cancers. Science.

[15] Bullock A.N., Henckel J., DeDecker B.S., Johnson C.M., Nikolova P.V., Proctor M.R., Lane D., Fersht A.R. (1997). Thermodynamic stability of wild-type and mutant p53 core domain. Proc. Natl. Acad. Sci. USA.

[16] Köbel M., Ronnett B.M., Singh N., Soslow R.A., Gilks C.B., McCluggage W.G. (2019). Interpretation of P53 Immunohistochemistry in Endometrial Carcinomas: Toward Increased Reproducibility. Int. J. Gynecol. Pathol..

[17] Bosari S., Viale G., Bossi P., Maggioni M., Coggi G., Murray J.J., Lee A.K.C. (1994). Cytoplasmic accumulation of p53 protein: An independent prognostic indicator in colorectal adenocarcinomas. J. Natl. Cancer Inst..

[18] Colomer A., Erill N., Verdú M., Roman R., Vidal A., Cordon-Cardo C., Puig X. (2003). Lack of p53 nuclear immunostaining is not indicative of absence of TP53 gene mutations in colorectal adenocarcinomas. Appl. Immunohistochem. Mol. Morphol..

[19] Levin T.G., Powell A.E., Davies P.S., Silk A.D., Dismuke A.D., Anderson E.C., Swain J.R., Wong M.H. (2010). Characterization of the intestinal cancer stem cell marker CD166 in the human and mouse gastrointestinal tract. Gastroenterology.

[20] Ginestier C., Hur M.H., Charafe-Jauffret E., Monville F., Dutcher J., Brown M., Jacquemier J., Viens P., Kleer C.G., Liu S. (2007). ALDH1 is a marker of normal and malignant human mammary stem cells and a predictor of poor clinical outcome. Cell Stem Cell.

[21] Huang E., Hynes M.J., Zhang T., Ginestier C., Dontu G., Appelman H., Fields J.Z., Wicha M.S., Boman B.M. (2009). Aldehyde dehydrogenase 1 is a marker for normal and malignant human colonic stem cells (SC) and tracks SC overpopulation during colon tumorigenesis. Cancer Res..

[22] Cheng J., Deng R., Zhang P., Wu C., Wu K., Shi L., Liu X., Bai J., Deng M., Shuai X. (2015). miR-219-5p plays a tumor suppressive role in colon cancer by targeting oncogene Sall4. Oncol. Rep..

[23] Dalerba P., Sahoo D., Paik S., Guo X., Yothers G., Song N., Wilcox-Fogel N., Forgó E., Rajendran P.S., Miranda S.P. (2016). CDX2 as a Prognostic Biomarker in Stage II and Stage III Colon Cancer. N. Engl. J. Med..

[24] Inaguma S., Lasota J., Wang Z., Felisiak-Golabek A., Ikeda H., Miettinen M. (2017). Clinicopathologic profile, immunophenotype, and genotype of CD274 (PD-L1)-positive colorectal carcinomas. Mod. Pathol..

[25] Huncharek M., Kupelnick B., Geschwind J., Caubet J. (2000). Prognostic significance of p53 mutations in non-small cell lung cancer: A meta-analysis of 829 cases from eight published studies. Cancer Lett..

[26] Iacopetta B. (2003). TP53 mutation in colorectal cancer. Hum. Mutat..

[27] Mitsudomi T., Hamajima N., Ogawa M., Takahashi T. (2000). Prognostic significance of p53 alterations in patients with non-small cell lung cancer: A meta-analysis. Clin. Cancer Res..

[28] Pharoah P.D.P., Day N.E., Caldas C. (1999). Somatic mutations in the p53 gene and prognosis in breast cancer: A meta-analysis. Br. J. Cancer.

[29] Guinney J., Dienstmann R., Wang X., De Reyniès A., Schlicker A., Soneson C., Marisa L., Roepman P., Nyamundanda G., Angelino P. (2015). The consensus molecular subtypes of colorectal cancer. Nat. Med..

[30] Hwang W.-L., Yang M.-H., Tsai M.-L., Lan H.-Y., Su S.-H., Chang S.-C., Teng H.-W., Yang S.-H., Lan Y.-T., Chiou S.-H. (2011). SNAIL regulates interleukin-8 expression, stem cell-like activity, and tumorigenicity of human colorectal carcinoma cells. Gastroenterology.

[31] Ladstein R.G., Bachmann I.M., Straume O., Akslen L.A. (2012). Prognostic importance of the mitotic marker phosphohistone H3 in cutaneous nodular melanoma. J. Investig. Dermatol..

[32] Nakashima S., Shiozaki A., Ichikawa D., Komatsu S., Konishi H., Iitaka D., Kubota T., Fujiwara H., Okamoto K., Kishimoto M. (2013). Anti-phosphohistone H3 as an independent prognostic factor in human esophageal squamous cell carcinoma. Anticancer Res..

[33] Nowak M., Svensson M.A., Carlsson J., Vogel W., Kebschull M., Wernert N., Kristiansen G., Andren O., Braun M., Perner S. (2014). Prognostic significance of phospho-histone H3 in prostate carcinoma. World J. Urol..

[34] Ramani P., Taylor S., Miller E., Sowa-Avugrah E., May M.T. (2015). High phosphohistone H3 expression correlates with adverse clinical, biological, and pathological factors in neuroblastomas. J. Histochem. Cytochem..

[35] Ribalta T., McCutcheon I.E., Aldape K.D., Bruner J.M., Fuller G.N. (2004). The mitosis-specific antibody anti-phosphohistone-H3 (PHH3) facilitates rapid reliable grading of meningiomas according to WHO 2000 criteria. Am. J. Surg. Pathol..

[36] Russo A., Russo G. (2017). Ribosomal Proteins Control or Bypass p53 during Nucleolar Stress. Int. J. Mol. Sci..

[37] Brierley J., Gospodarowicz M.K., Wittekind C. (2017). TNM Classification of Malignant Tumours.

[38] Kanda Y. (2013). Investigation of the freely available easy-to-use software ‘EZR’ for medical statistics. Bone Marrow Transpl..

