# Supplementary material for: The Complete Loss of p53 Expression Uniquely Predicts Worse Prognosis in Colorectal Cancer
Source: Int J Mol Sci. 2022 Mar 17;23(6):3252. doi: 10.3390/ijms23063252 (PMC8948732; doi:10.3390/ijms23063252)
Supplement: Supplementary file 1 [file ijms-23-03252-s001.zip › ijms-1613612-supplementary.pdf]

International Journal of
Molecular Sciences

MDPI

Supplementary Materials: The Complete Loss of p53 Expression Uniquely Predicts Worse Prognosis in Colorectal Cancer

Kazuhiro Nagao, Akira Koshino Akane, Sugimura-Nagata, Satoshi Inoue, Aya Nagano, Masayuki Komura, Akane Ueki, Masahide Ebi, Naotaka Ogasawara, Toyonori Tsuzuki, Kenji Kasai, Satoru Takahashi, Kunio Kasugai and Shingo Inaguma

SUPPLEMENTARY METHODS

Survival analyses using web site programs

The data from The Cancer Genome Atlas (TCGA) was analyzed using UCSC Xena program (https://xena.ucsc.edu/). The best cut-off values were automatically set by the program.

Supplementary Table S1. Antibodies and Conditions for Immunohistochemistry.

Genes	Reagent	Dilution	Antibodies
CCNA	IV	100	sc-751, Santa Cruz Biothechnology, Inc. (Dallas, TX)
CDX2	BM	25	DAK-CDX2, Dako/Agilent, (Santa Clara, CA)
GMNN	OV	500	EPR14637, Abcam (Cambridge, UK)
Ki-67	OV	100	Clone MIB-1, Dako/Agilent, (Santa Clara, CA)
MLH1	OV	200	Clone G168-728, BD Biosciences, (Franklin Lakes, NJ)
MSH2	OV	200	Clone G219-1129, BD Biosciences, (Franklin Lakes, NJ)
MSH6	OV	400	Clone 44/MSH6, BD Biosciences, (Franklin Lakes, NJ)
p53	BM	500	Clone DO7, Leica Biosystems (Wetzlar, Germany)
PMS2	OV+Linker	50	Clone A16-4, BD Biosciences, (Franklin Lakes, NJ)
PHH3	OV	500	Cell Marque™, Millipore SIGMA, (Rocklin, CA)

BM, Leica BondMax; IV, Ventana iView reagent; OV, Ventana OptiView reagent. Antigen retrieval was performed with heat activation in high pH buffer.

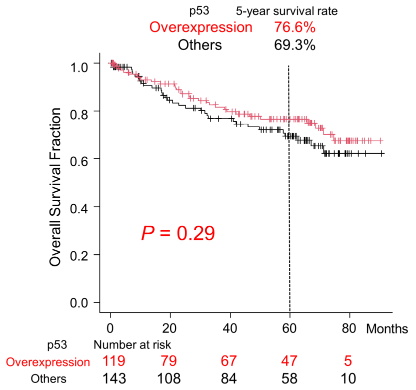

Supplementary Figure S1. Overall survival of CRC cases classified according to p53 immunophenotype. Kaplan-Meier curves for the patients grouped according to p53 expression patterns. Note that patients with CRCs with p53 overexpression tended to show better overall survival than others (wild-type, cytoplasmic expression and complete loss cases).

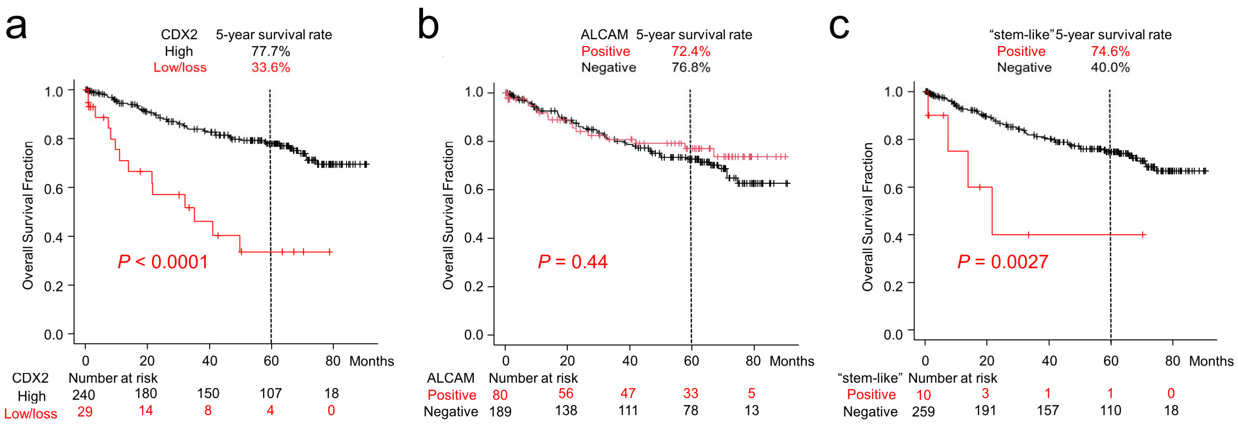

Supplementary Figure S2. Overall survival of CRC cases according to CDX2 and ALCAM expressions or "stem-like" immunophenotype. A to c, Kaplan-Meier curves for the patients grouped according to (a) CDX2, (b) ALCAM expressions and (c) "stem-like" immunophenotype. Note that CRC cases with decreased CDX2 expression ($P < 0.0001$) and cases with "stem-like" immunophenotype ($P = 0.0027$) showed significantly worse clinical outcome. ALCAM expression had no correlation to survival of CRC patients.

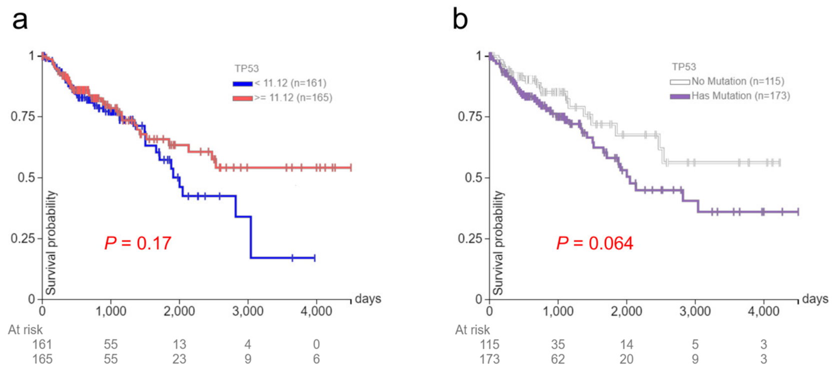

Supplementary Figure S3. Overall survival of CRC cases according to *TP53* expression or mutation status. a, Kaplan-Meier curves for the patients grouped according to *TP53* expression levels. Note that patients with CRCs expressing *TP53* at lower levels tended to showed worse overall survival. b, Kaplan-Meier curves for the patients grouped according to *TP53* mutation status. Note that patients with CRCs harboring *TP53* mutation tended to showed worse clinical outcome.
